# McNamara analysis cephalometric parameters in White-Brazilians, Japanese and Japanese-Brazilians with normal occlusion

**DOI:** 10.1590/2177-6709.26.1.e2119133.oar

**Published:** 2021-03-22

**Authors:** Juliana Moura STORNIOLO-SOUZA, Maria Pia SEMINARIO, Célia Regina Maio PINZAN-VERCELINO, Arnaldo PINZAN, Guilherme JANSON

**Affiliations:** 1 Universidade de São Paulo, Faculdade de Odontologia de Bauru, Departamento de Ortodontia (Bauru/SP, Brazil).; 2 Universidade Ceuma, Departamento de Ortodontia (São Luis/ MA, Brazil).

**Keywords:** Ethnic groups, Cephalometry, Diagnosis

## Abstract

**Introduction:**

McNamara’s Jr. cephalometric analysis is a tool to diagnose dental and skeletal discrepancies and is widely used, guiding diagnosis for surgical procedures to be performed or for the use of functional devices. Few studies have shown that different ethnic groups have different cephalometric patterns. Thus, single characteristics should be respected to support the diagnosis and to help the treatment plan for different ethnic groups and their different patterns of miscegenation.

**Objective:**

Obtain normal values for McNamara’s cephalometric analysis for adolescent Japanese-Brazilian descents with normal occlusion, as well as to compare this sample with similar samples of White-Brazilian and Japanese.

**Methods:**

Lateral headfilms from 40 White-Brazilian, 33 Japanese and 32 Japanese-Brazilian descents were selected. The three groups were composed by individuals with normal occlusion, well-balanced profiles and were separated by sex. The data were statistically analyzed with ANOVA, *t*-test, ANCOVA and MANCOVA tests.

**Results:**

White-Brazilian males had significantly greater nasolabial angle than Japanese males. Japanese-Brazilian displayed an intermediate value between White-Brazilian and Japanese.

**Conclusion:**

White-Brazilian, Japanese and Japanese-Brazilian present different cephalometric characteristics of McNamara analysis. Japanese males have a significantly more acute nasolabial angle than White-Brazilian subjects.

## RESUMO

**Introdução:**

A análise cefalométrica de McNamara Jr. é uma ferramenta amplamente utilizada para o diagnóstico das discrepâncias dentárias e esqueléticas, principalmente porque fornece um diagnóstico que orienta os procedimentos cirúrgicos a serem realizados ou a utilização de aparelhos funcionais. Estudos têm demonstrado que diferentes grupos étnicos apresentam padrões cefalométricos distintos. Portanto, as características próprias dos indivíduos devem ser respeitadas, para suportar o diagnóstico e facilitar o plano de tratamento, entre as diferentes etnias e seus diferentes padrões de miscigenação.

**Objetivo:**

Determinar os valores médios de normalidade das grandezas cefalométricas relacionadas à análise de McNamara em jovens mestiços nipo-brasileiros, com oclusão normal, descendentes de japoneses e brasileiros, e comparar os resultados das variáveis com amostras de jovens brasileiros leucodermas e xantodermas que apresentem as mesmas características.

**Métodos:**

Foram selecionadas 40 telerradiografias de jovens leucodermas, 33 de xantodermas e 32 de nipo-brasileiros. As três amostras foram constituídas de indivíduos com oclusão normal e face bem balanceada, e foram divididas por sexo. Os dados foram estatisticamente analisados utilizando ANOVA, teste *t*, ANCOVA e MANOVA.

**Resultados:**

Os leucodermas apresentaram o ângulo nasolabial significativamente maior, comparados aos japoneses, no sexo masculino; enquanto os nipo-brasileiros apresentaram um valor intermediário entre os leucodermas e xantodermas.

**Conclusão:**

Os grupos de leucodermas, xantodermas e nipo-brasileiros apresentaram diferentes características cefalométricas da análise de McNamara. Os xantodermas no sexo masculino tiveram o ângulo nasolabial significativamente mais agudo, quando comparados com os leucodermas.

## INTRODUCTION

In Orthodontics, several resources are available to aid diagnosis and the choice of treatment.^1^ ^-^ ^4^ Lateral headfilms, for example, have been very useful in determining a more precise diagnosis, because radiograph images can now be reproduced with a minimum of distortion, facilitating orthodontic evaluation. 

In 1941, Margolis^5^ presented an evaluation method that allowed the study of the facial outline, the mandible and the facial bones and their relationship with the teeth before and after orthodontic treatment, using cephalometric lateral headfilms and a profile photograph. In 1943, he verified that the incisor mandibular plane angle (IMPA) had to be 90º in a pleasant skeletal pattern.^3^

Nowadays, it is known that cephalometric analysis does not serve as a fixed and unique parameter for the examination of an individual and in the determination of orthodontic diagnosis and planning.^6^

McNamara Jr. analysis is widely known among orthodontists.^7^ It has some advantages over other analyzes, such as linear evaluations of apical base discrepancy and dental to apical base discrepancies. This analysis assists in the diagnosis and treatment planning of orthopedic or surgical cases. 

Many of the studies on orthodontic cephalometric analyzes were based on samples of North American individuals, of essentially Anglo-Saxon origin. Thus, in view of the vast miscegenation of the world population, there is a need for research to adapt the cephalometric values found in White-Brazilians to different ethnic groups.

Population migration between different countries increases each year and, currently, Brazil is the country with the largest number of Japanese outside Japan. Based on that, it is extremely important to differentiate the variations that dento-skeletal structures may present due to the union between different ethnic groups. Because of the considerable increase of the Japanese-Brazilian population, there is a need for studies to support the diagnosis and facilitate the treatment plan for these group. 

Therefore, the main purpose of this study is to compare three ethnic groups, White Brazilian, Japanese and Japanese-Brazilian, regarding the parameters of McNamara’s cephalometric analysis.

## MATERIAL AND METHODS

This study was approved by the Ethics in Research Committee of *Universidade de São Paulo* (USP, Brazil), under protocol number 080/2009.

The sample selection criteria included pleasing and balanced profiles with passive lip competence, facial symmetry, presence of normal occlusion or incipient Angle Class I malocclusion with normal overjet-overbite, absence or a minimal crowding of less than 2mm, presence of all permanent teeth (except the third molars), as judged using study models.

In order to confirm the absence of racial miscegenation, the grandparents and parents of the White-Brazilian and Japanese samples should not have had any miscegenation. The Japanese-Brazilian sample should be descendant of both Brazilians and Japanese, without previous miscegenation.

The White-Brazilian sample comprised 40 lateral headfilms of 20 females (mean age 13.70 ± 0.87 years) and 20 males (mean age 13.57 ± 1.03 years), from the files of the Orthodontic Department at *Faculdade de Odontologia de Bauru, Universidade de São Paulo*.

The Japanese sample consisted of 33 lateral head films of 17 females (mean age 15.65 ± 2.45 years) and 16 males (mean age 15.56 ± 2.51 years) whose parents and grandparents were born in Japan (excluding Okinawa Island).

The Japanese-Brazilian sample was constituted by 32 lateral headfilms of 17 females (mean age 13.22 ± 1.04 years) and 15 males (mean age of 14.79 ± 1.01 years). The inclusion criteria were the same as the other groups but in this case, all had to be children or grandchildren from the union of Japanese and White-Brazilians.

### CEPHALOMETRIC ANALYSIS

The lateral headfilms were taken with the teeth in maximum intercuspation, in accordance with the norms of the Discipline of Radiology of the institution.

All radiographs were traced by hand on acetate paper sheets of 17.5*x*17.5 cm, with 0.5-mm pencil, and a semi-rigid polypropylene black molding adapted to the negatoscope with the objective of concentrating the light to promote accurate visualization of the anatomical areas of interest.

**Figure 1:**
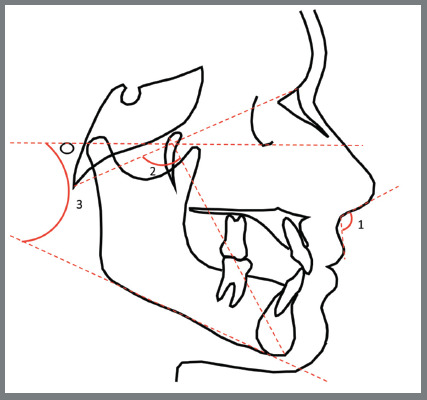
Angular measurements: 1) Nasolabial angle (Prn’.Sn.Ls); 2) Facial axis angle (BaN.PtGn); 3) Mandibular plane angle (PoOr.GoMe). Source: McNamara Jr,^7^ 1984.

**Figure 2:**
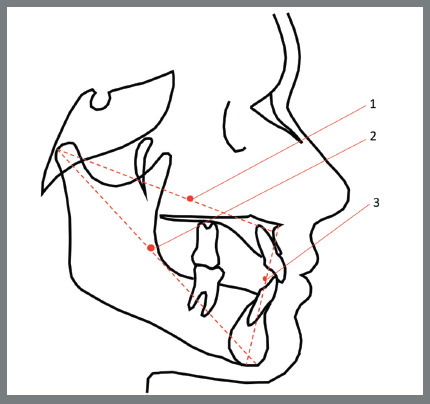
Reference lines and planes: 1) Midfacial length (Co-A); 2) Mandibular length (Co-Me); 3) Lower Anterior Facial Height (ANS-Me). Source: McNamara Jr,^7^ 1984.

**Figure 3:**
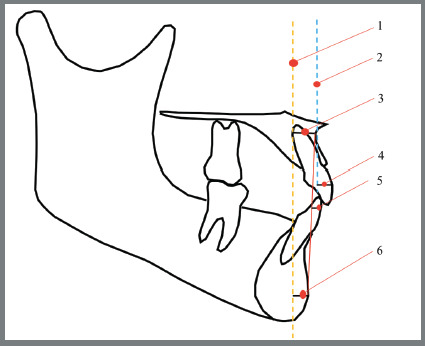
Reference lines: 1) Nperp, 2) Aperp, 3) A-Nperp, 4) Upper incisor to Point A vertical, 5) Lower incisor to A-Po line, 6) P-Nperp. Source: McNamara Jr,^7^ 1984.

The McNamara Jr. cephalometric variables were identified according to Table 1. The variables constructed with these landmarks are illustrated and described in Figures 1 to 3.

**Table 1:** McNamara cephalometric analysis variables.

Maxilla to cranial base	
A-Nperp	Distance between point A to Nasion perpendicular
Mandible to maxilla	
Mandibular length	Distance between Co and Gn
Midfacial length	Distance between Co and A
Maxillo-mandibular difference	Difference between the mandibular and maxillary lengths (Co-Gn) - (Co-A)
Lower Anterior Facial Height	Distance between ANS and Me
Mandibular plane angle	Angle between the anatomic Frankfurt horizontal plane and the line drawn along Go and Me (mandibular plane angle)
Facial axis angle	Angle formed by the line constructed from the pterigomaxillary fissure to Gn and the line constructed from Ba to point N
Mandible to cranial base	
P-Nperp	Distance between Pogonion and Nasion perpendicular
Dentition	
Upper incisor to Point A vertical	Distance between the facial surface of upper incisor to the perpendicular to Frankfurt plane, through point A
Lower incisor to A-Po line	Distance between the facial surface of mandibular incisor to the A-Po line
Soft tissue	
Nasolabial angle	Angle formed between lines Prn’-Sn and Sn-Ls

Subsequently, the landmarks were digitized with a Numonics, AccuGrid A30TL digitizer (Numonics Corporation, Montgomeryville, PA, USA), and stored in a computer, by a single investigator. The variables were measured with Dentofacial Planner 7.02 software (Dentofacial Planner Software Inc., Toronto, Ontario, Canada).

### METHOD ERROR

In order to evaluate the method error, 30% of the sample was randomly selected and re-measured after a 30-day interval by the same investigator. To estimate the random errors, the formula proposed by Dalberg^8^ (S^2^ = Σd^2^/2n, where S^2^ is the error variance and “d” is the difference between two determinations of the same variable) was applied. Systematic errors were evaluated with dependent *t*-tests, at *p*< 0.05.^9^

### STATISTICAL ANALYSES

Normal distribution of the variables was evaluated with Kolmogorov-Smirnov tests. All variables presented normal distribution.

Sexual dimorphism within each ethnic group was evaluated with *t*-tests. Ethnic intergroup comparison within males and females was performed with ANCOVA, using the age as co-variable, followed by Tukey tests and multivariate analysis of covariance (MANCOVA).

## RESULTS

The random errors ranged from 0.26mm (Lower incisor to A-Po line) to 0.74 mm (P-Nperp), and from 0.63º (Facial axis angle) to 1.6º (Nasolabial angle), and were within acceptable ranges.^10,11^ There were no significant systematic errors.

The Japanese sample was significantly older than the Japanese-Brazilian sample (Table 2).

**Table 2:** Intergroup age comparison between White-Brazilian, Japanese and Japanese-Brazilian and between males and females in the same ethnic group (Anova followed by Tukey tests).

	White- Brazilian		Japanese		Japanese-Brazilian		*p*
	Mean	SD	Mean	SD	Mean	SD	
Age	13.48^A^	0.89	15.30^A^	1.37	13.32^B^	1.44	0.000*
White-Brazilian							
	Females		Males				
Age	Mean	SD	Mean		SD		0.397
	13.56	0.81	13.40		0.97		
Japanese							
Age	Mean	SD	Mean		SD		0.830
	15.28	1.43	15.32		1.35		
Japanese-Brazilian							
Age	Mean	SD	Mean		SD		0.647
	13.05	1.51	13.64		1.33		

* Statistically significant at *p*< 0.05. Different superscript letters show significant differences between the means, by the Tukey test.

**Table 3:** White-Brazilian inter-sex comparison (*t*-tests).

White-Brazilian sample					
Variable	Female (n=20)		Male (n=20)		*p*
	Mean	SD	Mean	SD	
Maxilla to cranial base					
A-Nperp	0.24	3.57	-0.77	3.33	0.365
Mandible to maxilla					
Mandibular length (Co-Gn)	111.43	4.03	112.35	5.38	0.544
Midfacial length (Co-A)	85.53	3.22	87.00	4.45	0.237
Maxillo-mandibular difference	25.29	3.19	25.35	3.95	0.619
Lower Anterior Face Height (ANS-Me)	62.45	3.66	63.45	3.62	0.391
Mandibular plane angle (PoOr.GoMe)	24.25	4.99	26.52	3.87	0.116
Facial axis angle (BaN.PtGn)	90.41	3.07	90.98	2.35	0.514
Mandible to cranial base					
P-Nperp	-2.06	6.59	-4.49	4.59	0.185
Dentition					
Upper incisor to Point A vertical	4.24	1.62	3.65	2.20	0.340
Lower incisor to A-Po line	2.43	1.67	2.19	2.01	0.690
Soft tissue					
Nasolabial angle (Prn’.Sn.Ls)	105.71	7.46	106.72	10.56	0.727

Japanese males presented significantly greater maxillary retrusion, mandibular and midfacial lengths, and maxillomandibular difference than females (Table 4).

**Table 4:** Japanese inter-sex comparison (*t*-tests).

Japanese sample					
Variable	Female (n=17)		Male (n=16)		*p*
	Mean	SD	Mean	SD	
Maxilla to cranial base					
A-Nperp	-0.05	2.37	-2.09	2.28	0.017*
Mandible to maxilla					
Mandibular length (Co-Gn)	110.16	3.28	115.08	4.44	0.001*
Midfacial length (Co-A)	84.08	2.90	86.54	3.64	0.039*
Maxillo-mandibular difference	26.08	2.99	28.55	3.80	0.046*
Lower Anterior Facial Height (ANS-Me)	63.27	6.55	66.36	3.90	0.112
Mandibular plane angle (PoOr.GoMe)	27.39	6.88	26.89	5.28	0.817
Facial axis angle (BaN.PtGn)	89.26	4.02	88.23	3.22	0.426
Mandible to cranial base					
P-Nperp	-6.02	6.55	-6.88	4.18	0.661
Dentition					
Upper incisor to Point A vertical	3.82	1.87	4.63	2.23	0.271
Lower incisor to A-Po line	3.68	1.77	3.52	1.57	0.781
Soft tissues					
Nasolabial angle (Prn’.Sn.Ls)	101.12	10.49	96.16	7.61	0.132

* Statistically significant at *p*< 0.05.

Japanese-Brazilian males presented significantly greater lower anterior face height and mandibular retrusion than females (Table 5).

**Table 5:** Japanese-Brazilian inter-sex comparison (*t*-tests).

Japanese-Brazilian sample					
Variable	Female n=17		Male n=15		*p*
	Mean	SD	Mean	SD	
Maxilla to cranial base					
A-Nperp	0.86	1.88	-1.33	3.97	0.051
Mandible to maxilla					
Mandibular length (Co-Gn)	111.46	5.59	114.99	7.49	0.139
Midface length (Co-A)	84.22	3.05	86.83	5.49	0.102
Maxillo-mandibular difference	27.24	4.38	28.13	5.18	0.601
Lower Anterior Facial Height (ANS-Me)	62.36	2.58	66.89	5.10	0.003*
Mandibular plane angle (PoOr.GoMe)	23.85	3.00	27.16	6.11	0.057
Facial axis angle (BaN.PtGn)	91.10	2.53	88.93	4.54	0.099
Mandible to cranial base					
P-Nperp	-0.58	4.39	-6.05	6.95	0.011*
Dentition					
Upper incisor to Point A vertical	5.36	2.23	4.77	2.29	0.466
Lower incisor to A-Po line	2.89	2.20	3.66	2.49	0.363
Soft tissues					
Nasolabial angle (Prn’.Sn.Ls)	99.69	9.28	102.56	12.16	0.456

* Statistically significant *p*< 0.05.

There were significant ethnic intergroup differences among females (Table 6).

**Table 6:** Female intergroup comparisons (ANCOVA and Multivariate Test of Significance).

Variable	White-Brazilians (n=20)		Japanese (n=17)		Japanese- Brazilians (n=17)		*p*	*p*
	Mean	SD	Mean	SD	Mean	SD		
Maxilla to cranial base								0.009^†^
A-Nperp	0.24	3.57	-0.05	2.36	0.86	1.88	0.762	
Mandible to maxilla								
Mandibular length (Co-Gn)	111.43	4.03	110.16	3.28	111.46	5.59	0.073	
Midface length (Co-A)	85.53	3.22	84.08	2.90	84.22	3.05	0.148	
Maxillo-mandibular difference	25.92	3.19	26.08	2.99	27.24	4.38	0.165	
Lower Anterior Facial Height (ANS-Me)	62.45	3.66	63.27	6.55	62.36	2.58	0.728	
Mandibular plane angle (PoOr.GoMe)	24.25	4.99	27.39	6.88	23.85	3.00	0.170	
Facial axis angle (BaN.PtGn)	90.41	3.08	89.26	4.02	91.11	2.53	0.622	
Mandible to cranial base								
P-Nperp	-2.06	6.59	-6.02	6.55	-0.58	4.39	0.059	
Dentition								
Upper incisor to Point A vertical	4.24	1.62	3.82	1.87	5.36	2.23	0.183	
Lower incisor to A-Po line	2.43	1.67	3.68	1.77	2.89	2.20	0.080	
Soft tissues								
Nasolabial angle (Prn’.Sn.Ls)	105.71	7.46	101.12	10.49	99.69	9.28	0.074	

^†^ Statistically significant at *p*< 0.05 based on MANCOVA test.

White-Brazilians had significantly greater Nasolabial angle than Japanese and significant ethnic intergroup differences among males (Table 7).

**Table 7:** Male intergroup comparisons (ANCOVA followed by Tukey tests and Multivariate Test of Significance).

Variable	White-Brazilian (n=20)		Japanese (n=15)		Japanese-Brazilian (n=16)		*p*	*p*
	Mean	SD	Mean	SD	Mean	SD		
Maxilla to cranial base								0.049^†^
A-Nperp	-2.09	2.28	-0.77	3.33	-1.33	3.97	0.802	
Mandible to maxilla								
Mandibular length (Co-Gn)	112.35	5.38	115.08	4.44	114.99	7.49	0.786	
Midface length (Co-A)	87.00	4.45	86.54	3.64	86.83	5.49	0.764	
Maxillo-mandibular difference	25.35	3.95	28.55	3.80	28.13	5.18	0.336	
Lower Anterior Facial Height (ANS-Me)	63.45	3.62	66.36	3.90	66.89	5.10	0.195	
Mandibular plane angle (PoOr.GoMe)	26.52	3.87	26.89	5.28	27.16	6.11	0.927	
Facial axis angle (BaN.PtGn)	90.98	2.35	88.23	3.22	88.93	4.54	0.075	
Mandible to cranial base								
P-Nperp	-4.49	4.59	-6.88	4.18	-6.05	6.95	0.543	
Dentition								
Upper incisor to Point A vertical	3.65	2.20	4.63	2.23	4.77	2.29	0.311	
Lower incisor to A-Po line	2.19	2.01	3.52	1.57	3.66	2.49	0.111	
Soft tissues								
Nasolabial angle (Prn’.Sn.Ls)	106.72^A^	10.56	96.16^B^	7.61	102.56^A.B^	12.16	0.026*	

* Statistically significant at *p*<0.05 based on ANCOVA test. ^†^ Statistically significant at *p*<0.05 based on MANCOVA test. Different superscript letters show significant differences between the means by the Tukey test.

## DISCUSSION

### SAMPLE

Study samples have widely varied in the number of subjects, such as that of Cotton, Takano and Wong,^12^ who used a sample of only 20 individuals, and Shishikura,^13^ who used 132 individuals. Although the Japanese sample had a mean age significantly greater than the White-Brazilian and Japanese-Brazilian samples, in order to obtain a sample with the highest possible number of individuals, statistics was adjusted for the comparison between the three groups, in order to establish if the age would present any influence on the cephalometric measurements of the ethnic group.

Differences were found between male and female in the literature and in the present study.^11^ ^,^ ^14^ ^,^ ^15^ For this reason, the ethnic group comparisons had to be separated by sex.

### SEX DIFFERENCE

In the White-Brazilian sample, no significant differences between males and females were found (Table 3). Nevertheless, males presented higher values, compared to females. Similar findings were reported by Miyajima et al.,^16^ indicating that the dentoskeletal variables in males and females with ideal occlusion tend to be larger in males.

There was significantly greater retrusion of the maxilla in males than in females in the Japanese sample (Table 4). These characteristics of the skeletal tissues were confirmed by previous research.^17^ The midfacial length (Co-A), mandibular length (Co-Gn) and the maxillo-mandibular difference were significantly larger in males than in females. Similar findings were reported by Miyajima et al.^16^ and Bronfman et al.,^18^ who noticed significant sexual dimorphism in the midface and in mandibular length. Because the midface and the mandibular length were larger, the maxillo-mandibular difference was also larger in males than in females.

The Japanese-Brazilian sample presented dimorphism for some skeletal variables, with the highest values found for males (Table 5). Males displayed significantly greater anterior facial height than females. Supporting the results obtained by Ioi et al.,^19^ who concluded that Japanese males have significantly longer faces than Japanese females. Also, males showed the mandible statistically more retruded than females. Similar values were obtained in the literature by Alcalde et al.^17^

### ETHNIC DIFFERENCES

The samples were separated by sex to obtain a more specific and useful cephalometric normative values of each ethnic group.^16^ ^,^ ^17^ ^,^ ^20^ ^-^ ^22^

In males and females, the lower anterior face height was not significantly different between the three samples, which corroborates with the results of Nezu et al.^23^ and Alcalde et al.^17^ (Tables 6 and 7).

No significant differences in the dentition variables between the three samples could be noticed (Tables 6 and 7). The results found by Sathler et al.^24^ also demonstrated no differences in the upper incisor position between the Japanese-Brazilian and Caucasian sample. However, findings of Miura et al.,^25^ Miyajima et al.,^16^ and Ioi et al.^19^ found that Japanese-Brazilians have greater protrusion of the mandibular incisors.

Among the studied samples, only the nasolabial angle in males was significantly more acute in Japanese than in White-Brazilian (Table 7). The Japanese-Brazilian presented an average value intermediate to the group of White-Brazilian and Japanese. These results coincide with Bronfman et al.^18^ and Miyajima et al.,^16^ that also observed a more acute nasolabial angle in Japanese.

McNamara Jr.^7^ reports that separate evaluation of soft and skeletal tissues usually leads to the same diagnosis, so a patient with an acute nasolabial angle also has a protruded maxila. In this study, the male Japanese group that presented more acute nasolabial angle also had the greatest maxillary protrusion, which has been previously observed.^24^ In addittion, Miyajima et al.^16^ concluded that Japanese presents greater lip protrusion. 

Results showed significant differences between White-Brazilian, Japanese and Japanese-Brazilian using MANCOVA test (Tables 6 and 7). This test compares the three ethnic groups comparing all the variables together, in order to find differences between them. Previous studies found differences between Caucasians and Japanese sample^16^ ^,^ ^18^ ^,^ ^24^ ^,^ ^25^ that confirm the present findings. Therefore, the orthodontist must be careful and individualize each ethnic group during diagnosis and treatment planning.^26^ ^,^ ^27^

## CONCLUSIONS

» White-Brazilian, Japanese and Japanese-Brazilian have, in general, similar cephalometric characteristics of McNamara Jr. analysis.
» Japanese males have a significantly more acute nasolabial angle than White-Brazilian subjects.
» The findings of these study support that one cephalometric parameter is not appropriate to application in different ethnic groups because of the intergroups differences observed.
